# Mechanotransduction in Development: A Focus on Angiogenesis

**DOI:** 10.3390/biology14040346

**Published:** 2025-03-27

**Authors:** Simona Alibrandi, Carmela Rinaldi, Sergio Lucio Vinci, Alfredo Conti, Luigi Donato, Concetta Scimone, Antonina Sidoti, Rosalia D’Angelo

**Affiliations:** 1 Department of Biomedical and Dental Sciences and Morphofunctional Imaging, University of Messina, Street Consolare Valeria 1, 98125 Messina, Italy; 2 Department of Biomolecular Strategies, Genetics, Cutting-Edge Therapies, Istituto Euro-Mediterraneo di Scienza e Tecnologia (I.E.ME.S.T.), Street Michele Miraglia 20, 90139 Palermo, Italy; 3 Neuroradiology Unit, Department of Biomedical and Dental Sciences and Morphofunctional Imaging, University of Messina, Street Consolare Valeria 1, 98125 Messina, Italy; 4 IRCCS Istituto Delle Scienze Neurologiche di Bologna, Street Altura 3, 40123 Bologna, Italy; 5 Department of Biomedical and NeuroMotor Sciences (DiBiNeM), Alma Mater Studiorum—University of Bologna, 40127 Bologna, Italy

**Keywords:** mechanotransduction, development, angiogenesis, blood–brain barrier

## Abstract

Cells respond to external mechanical cues and transduce these forces into biological signals. This process is known as mechanotransduction and requires a group of proteins called mechanosensors. This peculiar class of receptors include extracellular matrix proteins, plasma membrane proteins, the cytoskeleton and the nuclear envelope. These cell components are responsive to a wide spectrum of physical cues including stiffness, tensile force, hydrostatic pressure and shear stress. Among mechanotransducers, the Transient Receptor Potential (TRP) and the PIEZO family members are mechanosensitive ion channels, coupling force transduction with intracellular cation transport. Their activity contributes to embryo development, tissue remodeling and repair, and cell homeostasis. In particular, vessel development is driven by hemodynamic cues such as flow direction and shear stress. Perturbed mechanotransduction is involved in several pathological vascular phenotypes including hereditary hemorrhagic telangiectasia. This review is conceived to summarize the most recent findings of mechanotransduction in development. We first collected main features of mechanosensitive proteins. However, we focused on the role of mechanical cues during development. Mechanosensitive ion channels and their function in vascular development are also discussed, with a focus on brain vessel morphogenesis.

## Simple Summary

This review article aims to describe the main features of a specific type of proteins mainly exposed on the cell surface. These proteins share the mechanism by which they respond to external mechanical stimuli in a process called mechanotransduction. By this process, mechanosensitive proteins act as receptors, converting these mechanical cues into biological signals that are elaborated within the cell. Several biochemical functions are controlled by mechanical stimuli including vascular development. Therefore, the role of mechanosensitive proteins in development and blood vessel formation is extensively discussed. The development of brain vessels is also addressed.

## 1. Introduction

Mechanotransduction refers to the cell ability to respond to mechanical stimuli. Both exogenous and endogenous cues continuously act on plasma membrane, inducing the cell to generate specific biological responses. Generated biochemical signals are not uniform; rather they depend on several force properties such as direction, magnitude, duration. Extrinsic stimuli including shear stress, blood flow, hydrostatic pressure, tension and matrix stiffness usually originate in the extracellular environment. On the other hand, intracellular tension contributes to reciprocal cell–cell mechanical regulation. This tension arises from forces related to actin polymerization and myosin contraction, as membrane curvature, cytoskeletal protrusive and traction cues, and nuclear envelope deformation. As biological significance, all molecular events involved in this signal transduction result in the modulation of gene expression, post-translational modifications and subcellular protein redistribution and they are known as mechanotransduction [1,2,3].

This process is allowed by specific proteins that enable both structural and biochemical communication between the extracellular matrix (ECM) and the subcellular structures, like the membrane, cytoskeleton and nucleus. Proteins responsive to mechanical cues are known as mechanosensors and comprise ion channels, integrins and transcription factors. All these proteins are capable of changing their conformation or subcellular localization, when a force is applied [4]. However, in polarized cells also the primary cilium responds to mechanical stimuli [5]. The primary cilium controls cell shape and motility during development and differentiation, since embryo stage to adulthood. In addition, stiffness, adhesion and tension are dynamic cues that make mechanotransduction a spatio-temporal specific response. Therefore, several physiological and pathological cell processes, as tissue remodelling, axon guidance, adhesion, tissue repair, autophagy and cancer are controlled by mechanotransduction [6,7,8,9,10]. For this reason, mechanotransduction is often accompanied by changes in cell metabolism [11,12,13]. In the last years, knowledge about the role of mechanosensation in regulating cell metabolism has rapidly increasing. This review summarizes the main findings on the different mechanoreceptors and is conceived to discuss how mechanotransduction contributes to development. In particular, blood vessel morphogenesis is considered, with a focus on blood–brain barrier (BBB) and its property maintaining.

### 1.1. Mechanosensors and Their Classification

Mechanosensation is not limited to a single family of proteins. Several membrane receptors, cytoskeletal proteins and transcription factors exhibit mechanosensitive properties. However, main concerns are on ion channels and the way by these can be considered as mechanotransducers. For this reason, criteria to classify an ion channel a mechanosensor were proposed by Arnadóttir et al. [14] and include: (i) presence of a pore-forming subunit for rapid ion conductance; (ii) ability to gate upon tension application; (iii) altered conductance upon structural defects of the channel domains; (iv) mechanosensation in a non-mechanosensitive cell upon forced expression of the channel; (v) both gene and protein mandatory expression in the mechanosensitive cell; (vi) abolition of cell mechanosensation due to mechanochannel gene loss of function. As above mentioned, several membrane proteins have been surprisingly found to be responsive to mechanical cues. However, all mechanosensors act by changing their conformation when cues are applied. This conformational change results in a biochemical response within the cell [15]. In general, signal transduction following a mechanical cue can occur by two different mechanisms. The first one strictly depends on membrane lipid bilayer deformation, following force application. This modification is perceived by integral membrane proteins, including enzymes and ion channels. As consequence, they become active triggering biochemical responses [16]. The second mechanism is based on both physical and functional continuity between ECM and cytoskeleton. In this context, forces directly act on membrane proteins, further responsive to stimuli coming from adjacent cells. Then, cues induce conformational changes such as to modify protein interaction pattern [17]. In this way, mechanosensing is even perceived to the nucleus [18].

### 1.2. The ECM–Membrane–Cytoskeleton–Nucleus Axis in Mechanosensing

**Figure 1:**
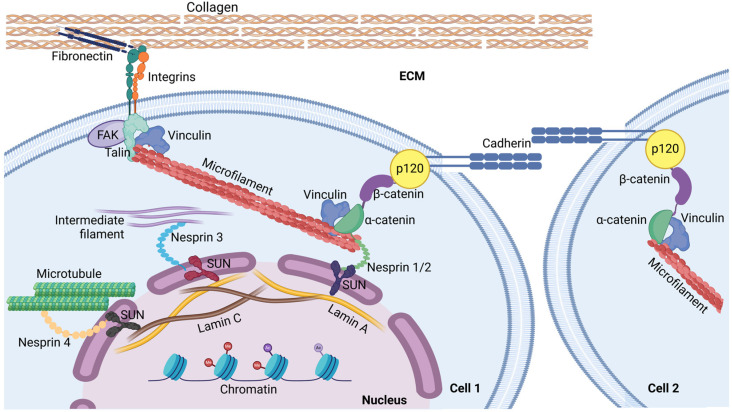
Mechanical signals from the ECM to the nucleus. Extracellular matrix mechanical cues are captured on cell surface by integrins and transmitted to cytoskeletal actin by the focal adhesion proteins. Likewise, tension from adjacent cell is transmitted by adherens junctions. Actin directly continues with nesprin 1/2, on nuclear envelope. By the SUN proteins, actin mechanical remodelling is trasmitted by the nesprins to the nuclear lamins, triggering chromatin remodelling and modification of the epigenetic pattern. ECM: extracellular matrix; FAK: focal adhesion kinase. Image created by the BioRender tool (https://www.biorender.com/).

Living cells are able to convert ECM signals into biochemical events. This requires a large number of proteins to make cells responsive. Extracellular matrix mechanical cues drive several processes as differentiation, proliferation, apoptosis, degeneration and malignancy through physical continuity among ECM, membrane proteins and cytoskeleton. Then, intracellular signals are further propagated within the nucleoplasm via the nuclear envelope [19]. Looking at this complex machinery, collagen, fibronectin, laminins are the main mechanical inducers in the ECM. Intracellular transmission is mediated by multimeric membrane receptors known as integrins. According to tissue distribution, their heterogeneous composition makes each cytotype differentially responsive to specific cues [20]. Other membrane proteins involved in mechanotransduction include receptors for extracellular soluble ligands, as transforming growth beta factor receptors (TGFβRs) or Wnt family members, and adhesion molecules [21,22,23]. Within the cell, stiffness and tension are transmitted to cytoskeleton by several integrin-associated proteins as vinculin, paxillin and talin [24]. In particular, talin was the first mechanosensitive cytoskeleton-associated protein, able to endure cues due to its structure. At the C-terminus, indeed, it contains 13 mechanosensitive rod subdomains that spread forces by switching between the unfolded/folded conformation [25]. By binding F-actin, talin transmits forces to the microfilament/myosin complexes that trigger contractility and, then, cell morphological change and protein subcellular redistribution. In this field, role of β-catenin was largely investigated. It usually localizes at adherens junctions (AJs) to stabilize cell–cell interaction. However, following mechanical stimulation, β-catenin is also able to translocate within the nucleus, where regulates expression of mechanosensitive genes [8,26]. It was shown that cytoskeletal cues are able to induce nuclear pore complex dilation, allowing nuclear shuttling of mechanosensitive transcription factors as the Krüppel-like Factor 2/4 (KLF), the Yes-associated protein (YAP) and the Transcriptional coactivator with PDZ-binding motif (TAZ) [27]. Adhesion proteins also participate in mechanotransduction. In particular, at AJs, proteins undergo conformational changes when a mechanical cue is applied; then, extracellular domains of the cadherins exposed on adjacent cells more strongly interact, tightening plasma membranes [28]. Likewise, their cytoplasmic tail binds actin. In this complex, the p120-catenin and the β-catenin contribute to cell junction stability [29]. The cytoskeleton responds to mechanical cues by further generating protrusive and traction forces. Traction requires myosin and allows intracellular signal transmission to ECM and neighbor cells [30]. Protrusive forces, instead, depend on actin polymerization and can result in filipodia formation, in certain cell types as neurons. Their dynamics mainly depends on extracellular ion concentration [31]. However, a recent study suggested that focal adhesions, actin orientation and intracellular force distribution are driven by cell morphology and, according to the adhesion curvature, they are able to pilot stem cell differentiation [32]. As mentioned, nucleus itself responds to forces by regulating envelope and nucleoskeleton remodelling, chromatin redistribution and histone modification. The Linker of Nucleoskeleton and Cytoskeleton (LINC) is the term coined to indicate this functional continuity between cytoskeleton and nucleoplasm. Structurally, LINC comprises two different protein families, nesprins and SUN proteins. By the cytoplasmic region, nesprins 1 and 2 bind actin, nesprin 3 binds intermediate filaments through the plectin, and nesprin 4 binds microtubule-associated proteins to be connected with microtubules. Nesprins span within the nuclear envelop where bind SUN proteins, that, in the nucleoplasm, are linked to the lamin A and lamin C. The SUN/lamin/chromatin complex is stabilized by emerin, acting as mechanosensor for the nucleus [33]. This mechanism is known as direct and it is such to lead heterochromatin redistribution at the inner nuclear membrane (Figure 1).

Conversely, another type of nuclear mechanotransduction called indirect depends on the monomer (G): polymer (F) actin ratio. Actin polymerization as response to certain mechanical cues, indeed, promotes nuclear shuttling of the Mixed-Lineage Kinase 1 (MLK1), a transcription factor targeting the serum response factor. In the absence of mechanical stimuli, MLK1 is retained in the cytoplasm through its binding to the G-actin [34]. Failed mechanotransduction at the nuclear envelope results in biogenesis of micronuclei, genome fragmentation and chromatin release into the cytoplasm [35]. This phenomenon usually occurs due to defects in LINC proteins. Laminopathies comprise more than 15 different phenotypes, as the Hutchinson–Gilford progeria syndrome [36] or the Greenberg dysplasia [37], arising due to mutations in lamin A (*LMNA*) and lamin B receptor (*LBR*) coding genes, respectively [38]. However, lamin dysfunctions have also been reported in cancer [39,40].

## 2. Mechanosensitive Ion Channels

Mechanosensitive ion channels (MCs) include heterogeneous membrane receptors activated by external mechanical cues. They show tissue-specific expression and selective responsiveness to specific stimuli. However, their common function is the intracellular ion flux following their activation. Mechanosensitive ion channels usually carry cations and they may be more or less selective [41]. The most characterized families include the epithelial sodium channel (ENaC) family, the TWIK-Related K^+^ (TREK) channels, the Big Potassium (BK) channels, the G-Protein Coupled Receptors (GPCRs), the Transient Receptor Potential (TRP) superfamily and the Piezo channels (Table 1). The ENaC family comprises 4 members contributing to electrolyte balance in epithelial tissues by regulating Na^+^ reabsorption, following hormone stimulation or proteolytic cleavage [42,43]. However, they can act as mechanotransducers in sensory neurons as well as in artery endothelial cells (ECs), despite the tethering mechanism is still unclear [44]. The TREK channels are three mechanogated two-pore domain K^+^ channels activated by membrane tension and expressed in heart, smooth muscle cells and nervous system, in particular at nodes of Ranvier [45]. Large-conductance voltage- and Ca^2+^- activated K^+^ channels (BK channels) regulate cell excitability by controlling K^+^ flux and Ca^2+^ signaling. They are mainly expressed in muscle cells where they enhance repolarization, in kidney, in vascular muscle cells and in central nervous system (CNS) [46,47,48,49]. Pathological conditions related to BK channel impairment include epilepsy, ataxia, hypertension and erectile dysfunction [50]. Recently, several GPCRs were further described as mechanosensors responsive to cues as shear stress, vibration or mechanical stretch, in order to regulate cell adhesion [51].

In this review, we describe the TRP superfamily and the Piezo channels as pivotal in vasculature, the main focus of our discussion.

### 2.1. Transient Receptor Potential (TRP) Family Members

**Figure 2:**
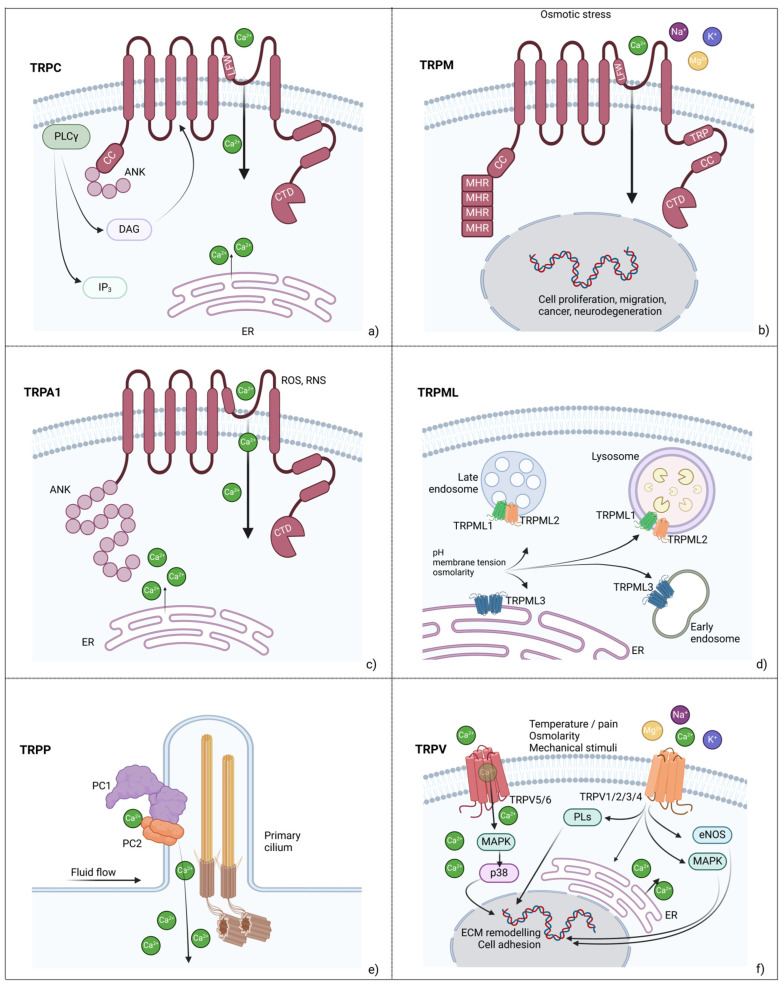
Mammalian TRP channels. The six mammal TRP channel classes include TRPC, TRPM, TRPA, TRPML, TRPP, TRPV. (**a**) TRPC channels show 4 ankyrin domains at the N-terminus that are recognized by the activated phospholipase C, upon stimulation. Downstream, intracellular calcium is mobilized. (**b**) TRPM channels exhibit 4 melastatin homology domains at the N-terminus; cation gating upon activation results in regulation of gene expression. (**c**) The TRPA1 channel has 16 ankyrin repeats at the N-terminus and contributes to calcium release from the endoplasmic reticulum. (**d**) The TRPML channels are expressed on endosomal and lysosomal membranes, forming either heterodimers (TRPML1/2) or homodimers (TRPML2/2). (**e**) The TRPP, also known as polycystins, are the most important mechanosensors of the primary cilium. (**f**) The TRPV channels are divided into two subclasses, the calcium-selective TRPV5/6 activate the MAPK-p38 cascade, regulating ECM remodeling and cell adhesion; the cation-aspecific TRPV1/2/3/4 activate the phospholipase cascades. ANK: ankyrin; CC: coiled-coil; CTD: carboxyl-terminal domain; DAG: diacyl-glycerol; ER: endoplasmic reticulum; IP3: inositol trisphosphate; MAPK: MAP-kinase; MHR: melastatin homology domains; eNOS: nitric oxide synthase; PC: polycystin; PL: phospholipase; RNS: reactive nitrogenous species; ROS: reactive oxygen species. Image created by the BioRender tool.

Transient receptor potential channels comprise several mechanosensitive cation channels regulated by phosphoinositides and firstly described as secondary mechanotransducers, amplifying Piezo channel-mediated primary mechanotransduction [69]. The TRP channels are classified into 7 large subfamilies, according to sequence homology. However, in mammals TRPs include the TRPC (canonical) subfamily made by 7 members, the TRPM (melastatin) subfamily made by 8 members, the TRPV (vanilloid) subfamily made by 6 members and a single TRPA (ankyrin) member. All these channels are mainly expressed in the cell membrane. The 3 TRPML (mucopilin) and the 3 TRPP (polycystins) receptors, instead, are expressed on the endoplasmic reticulum surface (Figure 2).

The TRPN (no mechanoreceptor potential C-like or NOMPC-like) subfamily has been identified only in fish and invertebrates. The TRP channels contribute to several physiological processes triggered by mechanotransduction, as cell cycle regulation, apoptosis, cell migration, chemo-osmotic stress response [70].

Briefly, the TRPC subfamily consists of Na^+^ and Ca^2+^-permeable nonselective cation channels particularly abundant in hippocampal neurons, where they play roles in memory, long-term potentiation, pain perception, anxiety [71]. With the exception of *TRPC2* that is a pseudogene [72], the 6 TRPC channel are expressed in humans and, according to structural homology, they are divided into 2 subgroups, TRPC 1/4/5 and TRPC 3/6/7. They are most often activated by phospholipase C (PLC) [73] and trigger cell membrane depolarization, contributing to store-operated Ca^2+^ entry (SOCE) [74]. However, phenotypes related to TRPC channel disfunction depend on impaired Ca^2+^ balancing and can affect several organs as kidney, muscle, heart, lung, pancreas [75,76,77,78]. Moreover, TRPC gain of function enhances cell proliferation, migration and invasion in cancer [79,80]. The TRPM subfamily comprises heterogeneous members most often regulating insulin and gastric hormone release [81,82]. Therefore, they are involved in several physio-pathological processes including proliferation and cell death, cancer and neurodegeneration [83,84]. The TRPM4 and TRPM5 channels are activated by Ca^2+^ but are only permeable to monovalent cations [85]. The TRPM7 is ubiquitously expressed and regulates Mg^2+^ homeostasis [86]. The TRPM2 acts as osmo-sensor and it was the first TRP channel identified as oxidative stress sensitive [87]. TRPM8 is expressed in sensory neurons where is activated by cold stimuli [88]. Finally, the TRPM3 is activated by heat and inflammatory pain [89].

The unique TRPA member (TRPA1) is a Ca^2+^ channel, contains 16 ankyrin repeats and is expressed in sensory neurons of skin and lung where it is responsive to mechanical stress, and in the brain where plays a role in nociception directly linked to migraine. It was initially described as a temperature sensor, being active when temperature is lower than 12 °C [90]. Endogenous Ca^2+^ itself regulates TRPA1 permeability and enhances cytoplasmic Ca^2+^ release from the endoplasmic reticulum [91]. TRPA1 is expressed in dendritic cells, endothelial cells, macrophages, neutrophils, mast cells, T-lymphocytes and chondrocytes where it responds to reactive oxygen and nitrogen species, irritants and proinflammatory cytokines [92]. Although it mainly triggers inflammation, its protective anti-inflammatory effect in both myocardial and brain ischemia was reported [93]. Furthermore, TRPA1 activation can also induce apoptosis and its dysfunction is associated with migraine, trigeminal nociception, glial inflammation in Alzheimer’s disease, inflammatory bowel disease, peripheral pain, lung disorders [94,95]. However, the rare autosomal dominant Familial Episodic Pain Syndrome (FEPS) arises following germline mutation in the *TRPA1* gene [96].

The TRPML subfamily comprises 3 members also known as mucolipins. Among the TRPs, these non-selective ion channels are the only ones expressed on endosomal surface, where they regulate membrane trafficking, vesicle pH and degradation and, then, autophagy [97]. They act as both homomeric and heteromeric complexes. In particular, TRPML1 and TRPML2 are expressed in late endosomes and lysosomes, homomeric TRPML3 is exposed on endoplasmic reticulum surface and early endosomes, while it forms heteromeric complexes in lysosomes. In addition to pH and osmolarity, mechanical tension generated by membrane tubulation also regulates TRPML activity [98]. The three TRPML proteins show different expression patterns, being TRPML1 ubiquitously expressed and, in particular, in brain, kidney, liver spleen and heart. Recessive loss of function mutations in *MCOLN1*, encoding for TRPML1, lead to mucolipidosis type IV (MLIV), a lysosomal storage disorder characterized by motor neurodegeneration, vision impairment and mental retardation, due to defective biogenesis of lipid-containing vacuoles [99,100]. TRPML3 is mainly expressed in sensory neurons, in the stria vascularis of the cochlea and in the organ of Corti sensory hair cells. Gain of function mutations, indeed, result in hearing loss and vestibular disorders [101]. Finally, TRPML2 expression is restricted to liver, kidney, spleen, heart and immune cells, where enhances viral endocytosis [102]. However, in immune cells as macrophages and natural killer cells, it acts as an antiviral, by promoting interferon release [103]. To date, no genetic diseases were linked to *TRPML2* mutations.

Polycystins are the three components of the TRPP subfamily. They were first discovered due to their association with the autosomal dominant polycystic kidney disease (ADPKD). All members are mechanosensitive Ca^2+^-permeable ion channels and, among these, PC1 and PC2 are the most well characterized. They are encoded by the *PKD1* and *PKD2,* respectively, and form heterocomplexes in the plasma membrane and in the primary cilium. In kidney primary cilium, PC1 is sensitive to urinary flow and its mechanoactivation triggers PC2, that acts as Ca^2+^ channel responsible for ion entry into the primary cilium and, then, into the cytoplasm [104]. This sequence of events controls tubule morphogenesis and defects of this mechanotransduction pathway result in ciliopathies, such as ADPKD [105]. Further, PC1 colocalizes with focal adhesions, where is sensitive to ECM stiffness, shear stress and adjacent cell tension. In this case, its activation results in a proteolytic cleavage by which the released peptide translocates to the nucleus, to directly regulate gene expression [106].

The TRPV or vanilloid subfamily is the most well characterized and it can be further subdivided into two groups. TRPV5 and TRPV6 are highly Ca^2+^-selective ion channels mostly expressed in epithelial cells, where they mediate Ca^2+^ entry in kidney and intestine, in physiological conditions. Germline mutations in *TRPV5* and *TRPV6* result in chronic kidney disease and nephrolithiasis, and in transient neonatal hyperparathyroidism, respectively [107]. TRPV1, TRPV2, TRPV3 and TRPV4 are non-selective cation channels mostly expressed in afferent neurons, where they function as somatosensors that detect temperature, nociceptive, mechanic and osmotic signals [108]. However, their expression was also confirmed in the endoplasmic reticulum, where they regulate Ca^2+^ homeostasis [109]. Among all TRPV members, only TRPV2 and TRPV4 have been confirmed as mechanosensors to date [110]. In particular, TRPV4 has been extensively studied due to its involvement in fibrillar collagen remodeling and ECM modulation, in accordance to its link with Charcot-Marie-Tooth disease type 2C and skeletal dysplasia [111,112]. *TRPV2* mutations, instead, cause heart failure [113]. Other TRPV-related phenotypes include migraine, neuropathic pain and dry eye disease (*TRPV1* gene) and Olmsted syndrome (*TRPV3* gene) [114,115].

### 2.2. Piezo Channels

**Figure 3:**
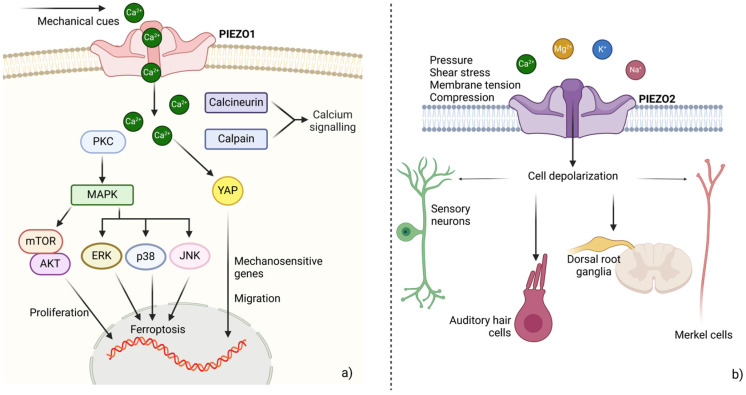
PIEZO channels. (**a**) PIEZO1 activation results in intracellular calcium influx; within the cell, protein kinase C can be activated. Downstream responses depend on stimulus properties and cell type. (**b**) PIEZO2 activation results in cation gating and cell depolarization. PKC: protein-kinase C. Image created by the BioRender tool.

Not so far, PIEZO1 and PIEZO2 were described as mechanically activated cation permeable channels, highly conserved in Vertebrates. In particular, PIEZO2 has nonselective conductance, while PIEZO1 is mainly Ca^2+^ selective [116]. They are very large proteins, located in plasma membrane, endoplasmic reticulum and nuclear membrane, differentially expressed across various cell types. During both embryo development and in adulthood they are activated by several stimuli including shear stress, membrane tension, cell compression. PIEZO1 is abundant in epithelial and endothelial tissues, bone and muscle, while PIEZO2 is largely expressed in sensory neurons, dorsal root ganglia and epithelial Merkel cells. However, both channels have baroceptor activity regulating blood pressure in the CNS [117]. Both the force-from-lipids and the force-from-filaments hypotheses have been proposed as mechanisms underlying PIEZO gating [118,119,120]. However, their biological activity is further modulated by several membrane proteins, as cadherins, TRPM4 and TMEM150C, which interact with PIEZO channels enhancing their function [121]; likewise, PECAM1, MTMR2 or PC2 act by reducing PIEZO activity [122,123,124] (Figure 3).

PIEZO1-mediated mechanosensing was initially described as a driver of EC polarity, in response to blood flow and as a key regulator of vascular remodeling [125,126]. However, it also responds to mechanical cues including membrane stretch and stiffness, tissue compression and distension [127,128,129,130]. Following activation, PIEZO1 opening increases intracellular Ca^2+^, that acts as second messenger [131]. PIEZO1 constitutive activation was shown to trigger mitochondrion-mediated apoptosis due to imbalanced Ca^2+^ homeostasis and, consequently, cytotoxic cation overload [132]. Likewise, dominant gain of function mutations result in hereditary xerocytosis, a hemolytic anemia characterized by primary erythrocyte dehydration [133]. In contrast, recessive loss of function mutations cause congenital lymphatic dysplasia [134] and, in particular, the lymphatic malformation-6 phenotype [135]. Finally, a novel *PIEZO1* missense mutation was recently shown to segregate within a family, together with the cerebral cavernous malformation (CCM) phenotype [136].

PIEZO2 is still poorly characterized. It was shown to be involved in auditory hair cell development [137]. Its expression abounds in sensory neurons, where triggers action potential and depolarization, following mechanogating [138]. Dominant phenotypes linked to *PIEZO2* gain of function mutations include Gordon syndrome, Marden-Walker syndrome and distal arthrogryposis type 5 [139]. Neurosensory deficits and skeletal defects, instead, arise following loss of function mutations [140,141].

### 2.3. Intracellular Mechanotransduction: Receptors for Soluble Ligands as Mechanosensors

Beyond integrins, receptors for extracellular soluble ligands can be mechanically activated, suggesting how biochemical and mechanical signals integrate to induce expression of mechanosensitive genes. Among the activated receptors, TGF-β and Wnt family members are responsive to mechanical stimuli. In many cases, intracellular transduction pathways converge on activation of mechanosensitive genes, to adapt cell homeostasis in response to mechanical force. Among these, SMAD, β-catenin, YAP/TAZ, Klf2/4 and ERK proteins are the most involved. SMAD proteins are activated downstream to the TGF-β signal and their biological activity is essential from early embryo development [142]. In the field of mechanotransduction, the TGF-β signal is involved in cell differentiation and several studies confirmed how extracellular cues can over-activated it, resulting in stemness acquisition or fibrotic phenotype. Differentiation loss was reported in human embryonic stem cells, when the TGF-β/Activin/Nodal signaling is amplified under force application [143]. This cascade was further characterized in kidney, where the primary cilium responds to fluid shear stress, not exclusively through polycystin activation. To regulate glomerular filtration, indeed, also TGF-β1 responds to shear stress and activates SMAD2/3 signal in renal epithelial cells. This signal is antagonized by Notch4 and its perturbation results in fibrotic phenotypes [144]. More in detail, shear stress enhances the expression of ligands for TGF-β and activin receptors. Downstream, SMAD2/3 over-activation results in increased Snai1 and reduced Cdh1 expression, enhancing epithelial differentiation loss and stemness acquisition [145]. Likewise, primary corneal keratocytes can differentiate into myofibroblasts, when the focal adhesion kinase and the TGF-β1 signal are activated. However, also in this case, differentiation rate depends on matrix stiffness and, then, on signal intensity [146]. The focal adhesion kinase complex was also shown to be triggered by matrix stiffness in cardiomyocytes, during cardiac development. Downstream, a multitude of signaling pathways are activated to control cell fate [147]. In detail, the PI3K/AKT and p38/JNK pathways ensure the expression of positive regulators of cardiac morphogenesis. In concerto, the YAP/TAZ, the AKT/TSC2/mTOR and ERK1/2 pathways control proliferation rate [148,149,150]. In cardiomyocytes, expression of mechanosensitive genes is also promoted by the non-canonical Wnt5a/Wnt11 pathway, in presence of pressure overload. In this context, the signal involves the TEAD1-YAP cascade that results in cardiomyocyte failure, suggesting how Wnt5a over-activation due to excessive mechanical stimuli can result in heart contractile dysfunction [151]. Recently, Wnt5a over-expression was reported in human artery ECs, when exposed to disturbed flow. This resulted in over-expression of its receptor frizzled-4 (FZD4) and, downstream, in β-catenin activation, and pro-inflammatory cytokine biosynthesis, stress fiber formation, contractile and atheroprone phenotype acquisition. These results were not observed under normal flow, further suggesting the role of disturbed hemodynamics in vascular phenotypes [152]. Blood flow also controls atrioventricular valve morphogenesis in zebrafish embryo, as shown by Paolini et al. In detail, shear stress activates both Notch and Klf2 signaling in endocardial cells; here, dll4-positive endocardial cells undergo lateral inhibition by Notch, become able to migrate in ECM and respond to paracrine Wnt9a, further produced by the Erk5-Klf2-Wnt9a cascade, upon flow stimulation [153]. The Wnt/β-catenin pathway also controls epithelial-to-mesenchymal transition in liver cancer cells, in a matrix stiffness-dependent manner. In particular, stiffer matrices enhance the expression of the long noncoding RNA (lncRNA) *NEAT1*, notoriously involved in liver cancer progression. Its role as positive cell cycle regulator and epithelial-to-mesenchymal transition promoter was reported, following its association with increased PCNA, N-cadherin and vimentin expression [154,155]. Interestingly, it was recently shown that *NEAT1* overexpression can induce stemness via the canonical Wnt/β-catenin pathway, suggesting as ECM properties can influence liver cancer progression.

Finally, the ERK pathway is also activated within cells, when shear stress, compression or tension are applied. In epithelial cells, increased ERK phosphorylation was observed after activation of the epidermal growth factor receptor by stretching of adjacent cells, resulting in polarization and motile phenotype acquisition [156]. Likewise, tension was shown to induce myosin II-dependent ERK activation, enhancing stress fiber formation in human fibroblasts [157]. Upon tension application, ERK activation can occur by two different mechanisms. The first involves a change in cell shape directly triggered by the cue [158]; the second is linked to the activation of mechanosensitive receptors on the cell surface, as PIEZO1 [159,160].

Therefore, as briefly described, cell response to mechanical cues is not uniform, despite downstream activated pathways most often involve the same proteins. Clearly, force magnitude, duration, intensity and cell type are the main parameters that determine cell fate.

## 3. Mechanosensing in Embryo Development

Mechanical cues drive cell fate since early stages of embryo development, controlling gastrulation and left-right symmetry breaking, gamete specification, cardiovascular development, kidney and inner ear morphogenesis, neuron migration, hematopoiesis [161,162,163,164].

During gastrulation, ECM forces induce mesodermal specification by activating the VE-cadherin/β-catenin signal, in both Drosophila and zebrafish embryos, in an evolutionary conserved manner [165]. In detail, exposure of the Y654-β-catenin phosphorylation site results from the β-catenin-VE-cadherin interaction, upon mechanical stretching. Beta-catenin phosphorylation by Src42A kinase leads to its release from junctions and its shuttling within the nucleus, where activates genes controlling morphogenic movements during gastrulation and mesoderm induction [166]. In addition, Y654-β-catenin phosphorylation is required for *Brachyury* expression, which is involved in endothelial-to-mesenchymal transition (EndMT), during mesoderm specification [167]. Within the early somite mesoderm, at the anterior extent of the primitive streak, a group of ciliated cells forms the node, also called the left-right organizer, involved in left-right specification [168,169]. Ciliated cells are divided in central “pit” cells and “crown” cells, exhibiting motile and immotile cilia, respectively [170]. On the crown cell surface, Wnt5A-dependent planar cell polarity drives distribution of posteriorly slanting cilia that express the Pkd2 [171]. Under nodal flow, pkd2 binds pdk1l1 driving embryo asymmetry, according to flow direction, as demonstrated in Medaka fish [172,173]. Furthermore, *Pkd1l1* mutant mice exhibited an altered Nodal expression pattern, not restricted to the left side downstream of nodal flow, resulting in loss of left-right asymmetry [174]. On the left side, Nodal induces *Pitx2* expression that controls organogenesis [175]. On the other hand, Nodal signaling is inhibited by Dand5. On the left dorsal side, Pkd2 expression allows Ca^2+^ influx following immotile cilium stimulation, leading to Dand5 degradation and consequent Nodal signal activation [176]. Dand5 further antagonizes the bone morphogenetic protein 4 (BMP) that regulates asymmetric distribution of Pkd2 in nodal immotile cilia, as recently shown by Katoh et al. [177]. Moreover, during zebrafish embryo gastrulation, tight junctions (TJs) respond to actomyosin tension controlling the separation between the enveloping cell layer and the yolk syncytial layer [178].

**Figure 4:**
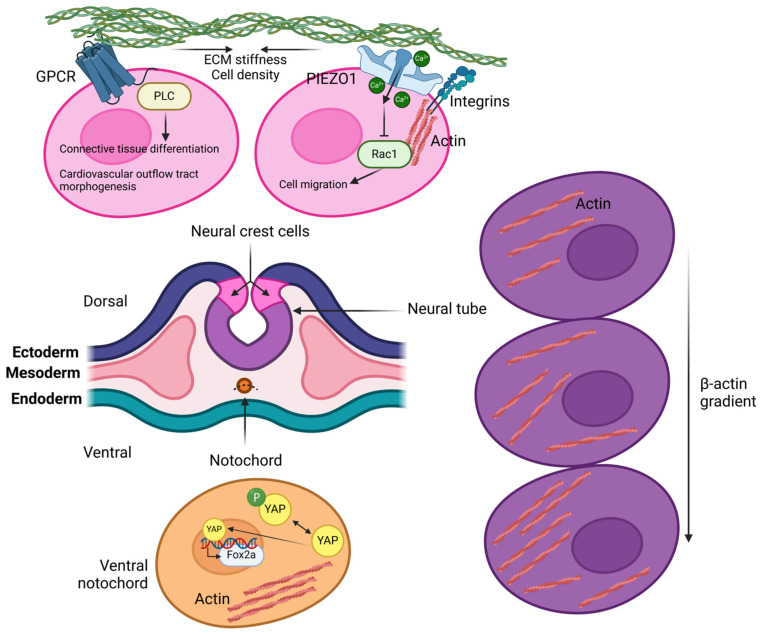
Mechanical regulation during embryo development. Extracellular matrix stiffness and cell density lead migration of neural crest cells; in particular, GPCRs and PIEZO1 drive mechanotransduction. Neural tube closure, instead, is driven by the β-actin gradient, increasing from dorsal to ventral cells. Likewise, differentiation of the cells of the ventral notochord region is driven by higher rigidity. ECM: extracellular matrix; GPCR: G-protein coupled receptor; PLC: phospholipase C. Image created by the BioRender tool.

Embryonic stem cell differentiation is controlled by ECM composition, elasticity and viscosity. For example, fibronectin expression controls axial mesoderm extension and migration, that are enhanced by the fibrillar form of the protein and its interaction with α5β1-integrin, in Xenopus [179,180]. Matrix stiffness is not uniform; rather its gradient depends on cell density, with stiffer areas hosting a higher cell number. Therefore, cells respond to this gradient by migrating from stiffer to softer regions, as shown by neural axon pathfinding during brain development in Xenopus embryo [181,182]. Cranial neural crest migration is mechanically controlled by other several factors including contact inhibition, rear actomyosin contraction and tissue fluidity [183]. However, together with migration, mesodermal stiffness also induces epithelial-to-mesenchymal transition of neural crest cells which remain undifferentiated until they reach their target tissue. Here, ECM stiffness controls neural crest lineage specification [184]. At the membrane level, neural crest cells express mechanosensitive GPCRs and ion channels. Among the GPCRs, the endothelin receptor, the sphingosine 1-phosphate receptor and the parathyroid hormone 1 receptor induce connective tissue differentiation [185,186,187]. Likewise, Piezo1 channel negatively controls cell migration [188] (Figure 4).

In addition, dorsoventral β-actin gradient controls neural tube closure and, in particular, higher tension is observed in the ventral neural tube cells, while greater rigidity occurs in the notochord ventral regions, where Yap activity is enhanced. This activates the Sonic Hedgehog pathway and, downstream, FoxA2 expression [189]. Further, Yap/Taz signaling guarantees proliferation of progenitor hindbrain boundary cells in zebrafish embryo, in response to mechanical cues [190]. In humans, neural stem progenitor cells respond to cell-generated traction forces by PIEZO1, but not PIEZO2, in a substrate stiffness-dependent manner. This results in neural stem progenitor cells differentiation promoting neuron lineage, against the astrocyte one [127]. Likewise, stiffer substrates induces neural crest differentiation into smooth muscle cells, while soft substrates result in acquisition of glial cell phenotype in mice [191]. Finally, ECM elasticity further controls adult mesenchymal stem cell (MSC) lineage specification in-vitro, and cell reprogramming depending on matrix composition is possible during early culture stage [192,193,194].

Musculoskeletal development is also controlled by mechanical stimulation [195,196]. Growth-generated strains lead early differentiation stages, while muscle loading occurs late during tissue development [197]. In the developing muscle, perichondral cells respond by generating forces that induce skeletal biogenesis [198]. At the same time, muscle contractile tension further promotes joint specification by inhibiting expression of Sox9 and Col2a1 chondrogenic markers. After specification, joint cavitation only partially depends on muscle tension [199]. On the other hand, loss of muscle contraction during tissue development perturbs *scxa* and *sox9a* expression in developing enthesis of zebrafish embryo [200]. More in detail, Scxa and Sox9 expression increases in mesenchymal stem cells, upon TGF-β2 activation, following mechanical stimulation. The intensity of TGF-β2 response depends on cue properties and results in downstream activation of SMAD proteins and Rho/ROCK/SRF signaling to control MSC differentiation in tenocytes [201]. Together with muscle and tendon, osteoblast mobilization for skeletal development was shown to depend on paracrine signals from ECs, in response to matrix stiffness and mechanical load, in a YAP/TAZ-dependent manner [202,203]. Finally, YAP activation by mechanical cues in smooth muscle cells is responsible for digestive tract elongation [204].

Knowledge about mechanical control of tissue morphogenesis is allowing to apply biomechanics to regenerative medicine. In the last decades, adhesion matrices have been engineered to drive stem cell differentiation and restore damaged tissues, such as osteonecrotic bone or infarcted myocardium [205,206]. It was shown that ECM properties lead stem cell lineage and these findings are allowing to modulate tissue regeneration in-vitro. For examples, increased gain of cartilage phenotype was observed in MSCs-loaded scaffolds, when grown in engineered chondrocyte pericellular matrix, able to trigger the TRPV4-YAP/TAZ-PI3K-Akt signaling within the cell [207]. Likewise, superparamagnetic iron oxide nanoparticles can induce mechanotransduction in human bone marrow-derived MSCs, leading to their osteogenic differentiation, upon external magnetic field application [208]. However, cells seem to maintain a mechanical memory, in response to prolonged persistence of the force. This mechanical memory includes both physical changes as cytoskeleton remodelling and nuclear deformation, and molecular events comprising epigenetic modifications [209,210]. Several factors contribute to mechanical memory acquisition and, among these, stiffness dose, time exposure and cell type [211]. Therefore, further knowledge in this field will allow to modulate mechanical memory and, then, cell plasticity [212,213].

## 4. Mechanotransduction in Vasculature

### 4.1. Mechanical Regulation of Endothelial Tip Cell Sprouting

**Figure 5:**
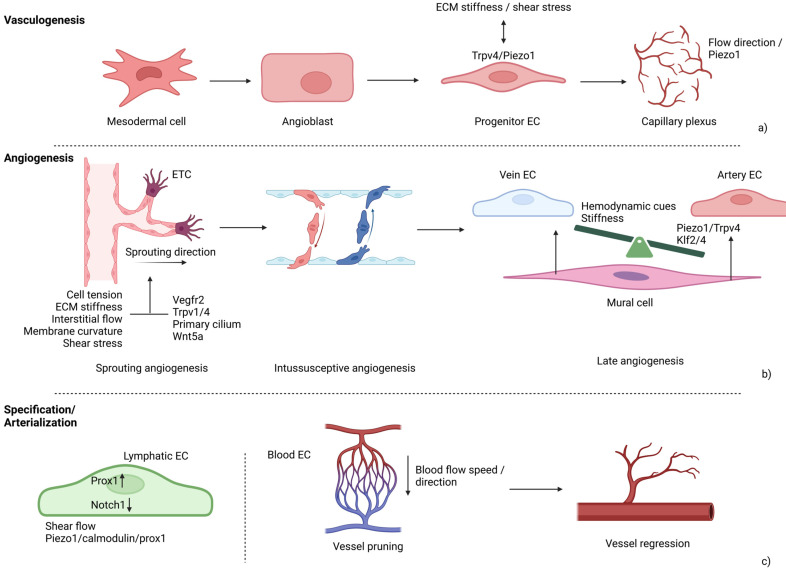
Mechanical regulation of vessel development. Vascular development can be divided into three moments: vasculogenesis, angiogenesis and specification. During vasculogenesis (**a**), mechanical stimuli generated by early blood flow act on progenitor endothelial cells and are mainly transduced by the TRPV4/PIEZO1 mechanosensitive ion channels, driving capillary plexus formation. During sprouting angiogenesis (**b**) both external and intracellular cues control endothelial tip cell protrusion. During late angiogenesis, mechanical properties of ECM surrounding mural cells control the early stages of arteriovenous differentiation. Finally, during specification (**c**), EC acquire their final identity as lymphatic ECs by expressing Prox1; likewise, blood flow velocity and direction drive vessel caliber during pruning. Finally, vascular regression contributes to the formation of the final vascular network. EC: endothelial cell; ECM: extracellular matrix; ETC: endothelial tip cell. Image created by the BioRender tool.

Vasculature morphogenesis begins already during early embryo development and continues throughout adulthood through remodeling phenomena. Correct vessel development requires integration of both biochemical and mechanical factors. Three main moments drive vessel network formation that, chronologically, are vasculogenesis, angiogenesis and arterialization [214] (Figure 5).

**Figure 6:**
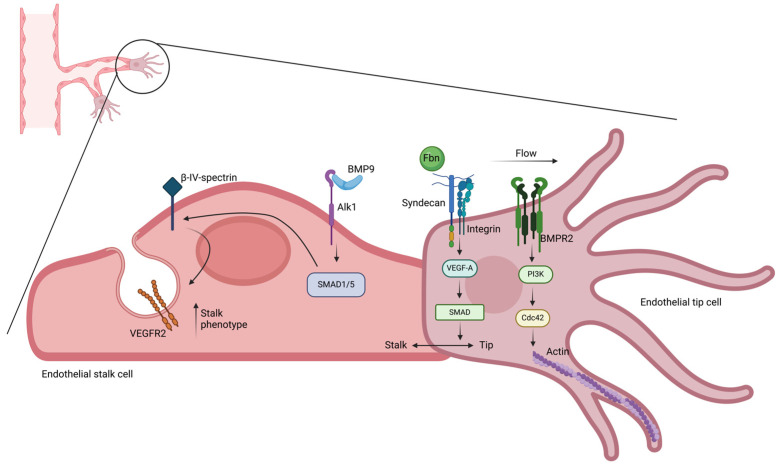
Mechanical signals in tip/stalk phenotype acquisition. At the angiogenetic front, during the angiogenic phase, EC can differentiate into either the stalk or tip phenotype. Tip cells exhibit by a protrusive behavior, driven by the BMPR2, activated by blood flow. Likewise, fibrillin binding to the integrin-syndecan complex directs differentiation toward the tip phenotype. In stalk cells, instead, the BMP9/Alk9 interaction promotes the β-IV-spectrin expression, responsible for VEGFR2 internalization. BMP: bone morphogenetic protein; BMPR2: bone morphogenetic protein receptor 2; FBN: fibrillin; VEGFR2: vascular endothelial growth factor receptor 2. Image created by the BioRender tool.

Vasculogenesis requires EC phenotype gain from angioblasts, previously differentiated from mesodermal cells. Endothelial progenitor cells are recruited and organized for de-novo formation of the primary capillary plexus [215]. From the primary capillary plexus, EC sprouting is promoted by both angiogenic factors and ECM remodeling. During this process, called angiogenesis, new vessels form from pre-existing capillaries and sprouting direction is driven by endothelial tip cells (ETCs) [216]. Endothelial tip cells are characterized by a protrusive phenotype, aimed to form transversal vessel connections. In contrast, endothelial stalk cells guarantee continuity with the original vessel from which sprout originates. Mechanistically, EC differentiation into the tip phenotype was recently shown to be controlled by several genes including *BMPR2* and *FBN1*. At the cell membrane, activated BMPR2 triggers the PI3K signaling to promote actin polymerization and filopodia formation, by the small Rho GTPase CDC42 [217]. At the angiogenetic front, fibrillin-1, encoded by the *FBN1* gene, transmits mechanical forces by binding integrins and syndecans [218]. Knock-out *Fbn1* mice exhibit retinal ETCs with disorganized cytoskeleton and reduced migration rate. In detail, fibrillin-1 deficiency was shown to cause impairment of the VEGF-A (vascular endothelial growth factor-A)/Notch/SMAD signal, leading to a compromised stalk-tip phenotype switch [219] (Figure 6).

Biochemically, ETCs show higher oxidative metabolism further enhanced by thicker matrices, due to increased energy demand required for sprouting in dense substrates [220]. Conversely, actin-mediated pulling in ETCs was demonstrated to induce ECM deformation during sprouting [221]. To balance stalk/tip differentiation, BMP9 binds Alk1, activates the SMAD1/5 pathway that induces β-IV-spectrin expression. In differentiating ECs, the β-IV-spectrin promotes vascular endothelial growth factor receptor 2 (VEGFR2) internalization, driving differentiation towards the stalk phenotype [222].

During sprouting angiogenesis, ECs undergo a collective migration program, responsible for the hierarchical vessel organization. In this context, the role of non-canonical Wnt pathway has been established. In particular, Wnt5a is activated by cell tension and stabilizes vinculin binding to α-catenin at adherens junctions, anchoring the cell membrane to the actin cytoskeleton [223]. Alternatively to sprouting, intussusceptive angiogenesis requires EC cytoplasmic protrusions within the lumen of a pre-existing small vessel [224]. At this stage, ECs begin to acquire their identity by expressing specific differentiation markers that drive cell specification. Differential expression of arterial, venous and lymphatic markers is flow-regulated, as shown in chick embryo [225]. However, together with ECs, also mural cells that are progenitors of both vascular smooth muscle cells (VSMCs) and pericytes, contribute to vessel specification as either large or small vessels. Interaction between endothelial and mural cells requires ECM proteins and soluble ligands, as fibroblast growth factor (FGF) and TGF-β, able to bind EC membrane receptors [226,227]. In this context, hemodynamic forces contribute to the endothelial phenotype by further recruiting VSMCs to cover vessels, according to their fate. Adhesion of VSMCs to ECs is encouraged by membrane receptors, including Notch members and chemotactic molecules as Semaphorin 3, released by the endothelium in response to hemodynamic cues. These data were obtained by Padget et al. in *Myl7^−/−^* mutant E10.5 yolk sacs, which exhibited reduced VSMC covering the arteries, when compared to wild-type embryos [228].

Finally, vascular remodeling requires both vessel pruning and regression. At this stage, ECs fully acquire their arterial or venous identity, organizing a hierarchical network. Pruning is driven by blood flow velocity, is characterized by EC migration and contributes to vascular hierarchy by controlling vessel caliber [229]. In this context, the role of the TGF-β co-receptor endoglin (ENG) was shown. Specifically, under blood flow, artery diameter is enlarged in *eng* zebrafish mutants and it depends on increased EC size. This suggests that endoglin acts as mechanosensor, regulating EC shape and volume in response to blood flow [230]. Regression, on the other hand, is based on programmed cell death and EC number reduction, resulting in vessel bed retraction [231].

### 4.2. Biomechanical Control of Angiogenesis

During angiogenesis, blood vessel development is driven by VEGF family members that bind to their receptors and, in particular VEGFR2, triggering its dimerization and phosphorylation [232]. However, on their surface, ECs also express mechanosensors that can be distinguished as apical or junctional. Apical mechanosensors include ion channels, GPCRs, the primary cilium, the glycocalyx and caveolae [233]. At cell junction, mechanotransduction relies on integrins, PECAM1, vascular endothelial cadherin (VEC) and VEGFR2 [234].

However, PECAM1/VEC/VEGFR2 and caveolae form a multimeric complex capable of responding to shear stress [235], resulting in activation of NADPH oxidase 2 (NOX2) and endothelial nitric oxide synthase (eNOS) [236,237,238,239]. In this context, VEGFR2 phosphorylation is enhanced by mechanical cues including interstitial flow, matrix stiffness, cell curvature and shear stress [240]. When activated, it dissociates from caveolin-1 and becomes phosphorylated [241]. Depending on the phosphorylation site, different downstream cascades involving mechanosensitive proteins can be activated. Among these, the MEK/ERK pathway activates the mechanosensitive transcription factors YAP/TAZ, resulting in a decreased EC proliferation rate, both in-vitro and in zebrafish [242]; the Rho/ROCK cascade leads to focal adhesion kinase activation, actin polymerization and stress fiber formation; the PI3K/Akt and the p38/MAPK pathways [243]. In particular, YAP/TAZ nuclear translocation is enhanced by stiffness and disturbed blood flow, and is mediated by the Dll4-NOTCH1 interaction [244]. NOTCH1 as mechanosensor was recently described [245,246], and it acts by downstream activating SMAD6, which helps maintain EC barrier integrity, under shear stress condition [247]. Moreover, the focal adhesion protein DLC1 was described as a transcriptional target of YAP/TAZ in ECs, and it was recently shown to reduce YAP nuclear localization during sprouting angiogenesis in human umbilical vein endothelial cells (HUVECs), in response to cytoskeletal tension [248].

Under laminar flow conditions, mechanosensing signals originate from ECM proteins and are transmitted to integrins. Downstream of integrin activation, the FAK-ERK1/2, PI3K-AKT-eNOS and Rho/ROCK cascades are activated promoting cell survival, proliferation and migration [249]. However, the key EC mechanosensitive transcription factors activated by ECM stimuli are YAP and TAZ. YAP activation and its nuclear translocation result in a hyperproliferative and pro-inflammatory EC phenotype, commonly observed in vessel pathological conditions, as atherosclerosis [250]. Proliferation depends on ERK1/2 phosphorylation rate that increases in ECs growing in stiffer substrates [251].

Together with ECM-derived cues, several hemodynamic forces act on ECs driving vessel development and remodeling, both in embryo and in adulthood. Their magnitude and duration differ according to developmental stage, body region and physio-pathological condition. Shear stress is the most studied. It is parallel to the endothelium and depends on vessel caliber, blood density and velocity. In response to shear stress, ECs rearrange cytoskeleton and enhance junction stability, by encouraging PECAM1-actin microfilament interaction. Together, these morphological changes allow EC polarization according to flow direction, and this phenomenon is predominant in arteries [252]. In addition, the pivotal role of vinculin localization in junctional fingers during lumen expansion was reported [253]. Upon extracellular mechanical cues, EC cytoskeleton reorganization is driven by interaction between VEC–catenin complex and actin to stabilize AJs and to maintain barrier properties. Vinculin knock-out zebrafish exhibited a more permeable blood barrier, although this effect was limited to small molecules [254].

Blood pressure, instead, influences the vasculature by generating circumferential and axial stresses, that act tangentially and longitudinally on the internal lumen, respectively [255]. Intraluminal cues further result in VSMC stretch and vessel diameter regulation. In particular, VSMCs respond to circumferential stress induced by high blood pressure by adopting a contractile phenotype, that results in vessel caliber reduction. Likewise, under lower blood pressure, VSMC response results in vessel dilatation. This behavior is known as vascular myogenic response, and the main players acting in this mechanotransduction include integrins, cadherins and GPCRs [256]. Recently, latrophilin-2 was described as a novel GPCR activator in ECs. It is activated by flow and triggers pro-angiogenic signals through PECAM1 [257].

Beyond hemodynamic forces, also hydrostatic pressure acts on ECs and enhances tube formation by activating the Ras/ERK pathway, in a VEGFR2-indepenent manner. In detail, hydrostatic pressure does not directly activate the GPCRs/PKC cascade in HUVECs. Rather, its activation occurs downstream of aquaporin-1 mediated water efflux, following cell membrane exposure to the cue [258]. Mechanical cues further modify EC membrane physical and chemical parameters including membrane fluidity, thickness and the lipid order, which refers to the lipid phase transition from a liquid-disordered state to a liquid-ordered state [259,260]. About membrane chemical composition, cholesterol quantity was shown to decrease in response to laminar shear stress [261,262].

As described, membrane proteins act as mechanosensors inducing vascular remodeling, upon activation by ECM components. Cyclic stretch was shown to enhance mural cells to release thrombospondin-1 that binds integrin αvβ1, on EC surface. Downstream, this results in actin stress fiber polymerization and in YAP nuclear translocation [263]. In a recent study Seetharaman and colleagues identified novel mechanosensitive proteins in ECs, responsive to flow, comprising the FHL2 transcription factor, known to be involved in atherosclerosis progression [264]. FHL2 is early activated when flow changes and is capable of binding actin stress fibers to modulate AJ force-adaptation [265]. Other focal adhesion components involved in mechanosensation include the Lin-11, lsl-1 and Mec-3 (LIM) domain-containing proteins, regulating cell motility, apoptosis and proliferation in response to hemodynamic cues. In particular, zyxin is enable to shuttle from focal adhesion to the nucleus, where binds transcription factors and regulates gene expression, promoting VSMC contractility, in response to stretch [266].

### 4.3. Mechanosensitive Ion Channels in Vascular Development

Nowadays, the role of Ca^2+^ homeostasis in the behavior of non-contractile cells is plentifully studied. Mechanosensitive channels expressed on the EC membrane contribute to intracellular Ca^2+^ balance. Downstream, the regulation of Ca^2+^ release from the endoplasmic reticulum is crucial for cell junction maintaining and vessel permeability, under shear stress condition or increased blood flow [267]. In this context, among the mechanosensitive receptors previously described, several TRP family members and PIEZO1 mainly response to stretching and membrane tension triggering pro-angiogenic responses. Their expression differs throughout the vasculature, according to species, anatomical region and vessel caliber [268].

In mammals, TRPC and TRPV channels consistently contribute to vascular function. As described, during sprouting angiogenesis, mechanical cues cooperate with VEGF to guide ETC pathfinding. In response to VEGF, TRPC1 induces filipodia formation and EC polarization, despite this receptor does not appear to be essential for tube formation [269,270]. Under oxidative stress, TRPC4 was shown to enhance VEGF release in coronary artery ECs [271], as well as it induces retinal neovascularization under hypoxic condition [272]. Together with VEGF, erythropoietin and thrombin can also activate EC mechanosensors and, in particular, the TRPC3, resulting in increased cell proliferation and migration rate [273]. Further, in ECs, zyxin nuclear translocation occurs downstream of TRPC3 activation and contributes to endothelial phenotype maintaining, under increased blood pressure conditions [266]. In addition, TRPC1 and TRPC4 are involved in SOCE; their activation triggers the phospholipase C signal with consequent Ca^2+^ release from the endoplasmic reticulum [74]. Finally, TRPC6 and TRPC7 control the receptor-operated calcium entry (ROCE) within the cell [274].

About the TRPV members, the TRPV1 and TRPV4 are the main angiogenic regulators further acting on VSMC function [275]. TRPV1 induces vascular relaxation by mediating both eNOS activation and ECM remodeling, in response to shear stress [276]. Although TRPV1 was initially detected in neurons, where regulates synaptic transmission linked to nociception, it is also highly expressed in perivascular nerves and peripheral endothelium of visceral organs as lung. In these tissues TRPV1 responds to oxidative stress and ischemia, suggesting its role in pulmonary arterial hypertension pathogenesis [277,278].

In VSMCs, activated TRPV1 inhibits neointima formation and promotes EC proliferation and migration in a mitofusin 2-dependent manner, involving mitochondrial homeostasis [279]. Also TRPV4 dysfunction can impair mitochondrial homeostasis. In detail, TRPV4 over-activation disrupts the bioenergetic function of mitochondria in pulmonary arterial ECs due to eNOS uncoupling. Additionally, it increases migration and proliferation rates in microvascular ECs [280,281].

It was shown that TRPV1 forms heterodimers with TRPV4, promoting intracellular Ca^2+^ influx and sprouting angiogenesis in a VEGF-independent manner, in retinal microvascular ECs [282]. In retinal ECs, TRPV4 further contributes to pathological neovascularization due to reduced pericyte coverage, in response to oxidative damage. However, its depletion has not effect under physiological conditions [283].

TRPV4 is expressed early in endothelial progenitor cells [284]. Its down-expression was described in endothelium of several tumors, where ECs appear immature, permeable and covered by a reduced number of pericytes. However, the phenotype ameliorates when VEC expression increases, upon TRPV4 activation by mechanical cues, suggesting that TRPV4 contributes to vascular integrity in response to ECM stiffness [285]. In ECs, TRPV4 is also activated by shear stress and induces neovascularization by promoting VEGFR2 expression. Notably, VEGFR2 phosphorylation increases following TRPV4 silencing due to YAP/TAZ nuclear translocation. Increased VEGFR2 phosphorylation results in its shuttling from the perinuclear region to the plasma membrane, suggesting its gain of function. When phosphorylated, VEGFR2 promotes EC migration and collateral vessel development during arterialization [286,287]. Moreover, it was recently shown as TRPV4 contributes to lung microvessel disfunction in cystic fibrosis patients. In detail, the cystic fibrosis transmembrane conductance regulator (CFTR) protein controls endothelial properties by interacting with PIEZO1 on EC surface; its deficiency impairs PIEZO1 activity, under shear stress condition. As consequence, altered lipid metabolism leads to increased TRVP4 expression with calpain signal amplification and, then, endothelial barrier failure [288]. Regarding PIEZO1, it is expressed in various cell types, particularly in bone, cardiac, kidney and brain endothelium. It responds to shear stress, enhancing Ca^2+^ intracellular entry. This results in EC polarization and alignment to the flow direction, through protease activation [289]. During embryo development, *PIEZO1* expression begins with blood circulation, becoming it abundant in mice ECs since E.9. Shear flow induces actin polymerization and stress fiber formation driving EC elongation and alignment. *Piezo1* knock-down was shown to impair EC behavior due to failed mechanotransduction. Moreover, defects of the early vascular plexus were demonstrated in *Piezo1* mutant mice, showing loss of large-diameter vessels [125].

In bone tissue, endothelial PIEZO1-mediated mechanotransduction enhances fracture repair by promoting vessel remodeling, to restore blood flow in damaged tissue. Conditional endothelial *Piezo1* loss of function, indeed, results in reduced intracellular Ca^2+^ concentration and failed calpain activation, as well as in reduced PI3K/AKT phosphorylation and NOTCH1 and PECAM1 down-expression. These findings suggest that *Piezo1* depletion in ECs can impair angiogenesis during bone repair, in open femoral shaft transverse fracture mice model [290].

Recently, Abello and colleagues described how blood flow regulates the expression of the mechanosensitive transcription factor klf2a in zebrafish, during vascular development. It drives arterial or venous differentiation, in response to pulsatile or laminar hemodynamic cues, respectively, by directing mural cell migration. More in detail, *klf2a* expression increases or decreases following piezo1 block or activation respectively, suggesting as piezo1 gating in response to shear stress contributes to the cross-talk between endothelial and mural cells [291]. EC behavior control by PIEZO1 depends on different shear stress and stiffness conditions. In detail, on a stiff substrate and under high shear stress, *PIEZO1* knock-down results in disoriented stress fiber polymerization and reduced cell and nucleus size. In contrast, on soft matrix, ECs appear biggest and with increased nucleus area, upon *PIEZO1* loss of function. Therefore, PIEZO1 drives stress fiber formation and controls cell shape by regulating cytoskeletal organization, according to flow dynamics and ECM rigidity [292]. PIEZO1 pro-angiogenetic function was further confirmed by Kang et al. The authors demonstrated that, following shear stress, PIEZO1-mediated Ca^2+^ influx modulates matrix metallopeptidase 2 (MMP2) activation to enhance membrane type 1-matrix metalloproteinase (MT1-MMP) translocation from the endoplasmic reticulum to the plasma membrane, to remodel ECM and to promote sprouting angiogenesis [293].

Finally, *PIEZO1* expression in mitochondria of ECs was recently shown. Here, its gating enhances glycolysis, oxidative metabolism and ATP synthesis, explaining how the endothelium responds to energy demand, under shear stress conditions [294].

### 4.4. Mechanotransduction in Lymphangiogenesis

Together with blood vasculature, also lymphatic vessels development is influenced by physical forces. The hierarchical organization of lymphatic tree starts at the tissue interstitium with small caliber vessels, called capillary lymphatics. These consist of a lymphatic EC monolayer lacking of mural cells, continue to collectors and, then, to the thoracic duct. During development, embryonic fluid drainage is sufficient to trigger lymphatic vessel sprouting in-vitro [295]. Exceptionally, under shear flow, lymphatic ECs show reduced Notch1 activity and enhanced sprouting. This is mediated by Orai1, a calcium channel involved in SOCE. Orai1 activity was recently shown to depend on Piezo1, that acts as primary flow mechanosensor [296]. In zebrafish, an increased intracellular Ca^2+^ results in calmodulin activation and its binding with prox1, key regulator of lymphangiogenesis [297]. By binding Klf2, the Prox1/calmodulin complex forms a trimeric structure that suppresses Notch1 expression, by upregulating Dtx1 and Dtx3l [298]. Likewise, Klf2 and Klf4 expression is induced by Orai1; downstream, they activate expression of *Vegfa*, *Vegfc*, *Fgfr3* and *Cdkn1c*, involved in lymphatic ECs proliferation [299].

Recently, Qin and colleagues demonstrated that medium stiff matrices, supplied with VEGF-C, are sufficient to promote sprouting lymphangiogenesis by enhancing the integrin/FAK mechanotransduction pathway, in cultured lymphatic ECs, suggesting new perspectives for lymphatic vessel regeneration [300].

## 5. Mechanosensing of the Blood-Brain Barrier

Unlike in other anatomical regions, the endothelium of the CNS is characterized by high selectivity, which is ensured by specialized cell junctions and metabolite transport strategies. Across the blood–brain barrier (BBB), molecules can move via three different mechanisms. Two of these require transporters, the ATP-binding cassette transporter (ABC) superfamily and the solute carrier (SLC) superfamily. The third mechanism is based on endocytosis and includes receptor-mediated transcytosis (RMT) and absorptive-mediated transcytosis (AMT). As the name suggests, the ABC transporters rely on ATP hydrolysis to move molecules from the ECs to the blood; the family includes about 50 members, divided into 7 families (ABCA to ABCG). The SLC channels facilitate passive transport of small molecules as ions, organic molecules, metabolites, neurotransmitters and xenobiotics. This superfamily include approximately 500 different transporters organized into 66 families (https://slc.bioparadigms.org/). They can be more or less selective and can mediate either influx or efflux of substances [301]. Finally, RMT requires a highly selective interaction between membrane receptors and macromolecules to be internalized; in contrast, AMT is responsible for cationic substrate absorption onto the caveolae surface. By these types of transcytosis, ECs transport peptides, antibodies, low-density lipoproteins and albumin [302]. These highly selective transcytosis mechanisms can occur from the brain to the bloodstream and vice versa [303]. However, despite these strategies, ECs alone are not sufficient to guarantee this barrier function; rather also pericytes, neurons, astrocytes and ECM contribute to vascular impermeability. For examples, pericytes regulate vascular leakage; likewise astrocytes and neurons release vasoactive molecules to modulate blood flow. For this reason, the term “blood–brain barrier” was coined by Paul Ehrlich, Edwin Goldmann and Lena Stern about a century ago. However, BBB properties are not uniform across different CSN areas, as they vary for capillary density, junction distribution, astrocyte and pericyte number [304]. These differences result from differential gene expression in specific brain areas [305]. Certainly, ECs are the most abundant cells of the BBB, anchored by both TJs and AJs, directly involved in selective permeability. Biogenesis of TJs during development was shown to be driven by VEC. In detail, AJ assembly occurs early during vascular development, as blood flow promotes VEC-mediated cell adhesion. Within ECs, this results in β-catenin stabilization at cell junctions, Akt-mediated FoxO1 phosphorylation and enhanced expression of claudin-5, a key component of TJs [306]. In addition, endothelial β-catenin disruption downregulates claudin-1 and claudin-3 expression in brain ECs, impairing TJ assembly [307]. By β-catenin, claudin-5 regulation is context-dependent, according to differential Wnt family member expression in brain regions. In particular, astrocytes are the primary source of extracellular secretion of Wnt ligands and the cytotype largely involved in generation of extracellular cues [308].

**Figure 7:**
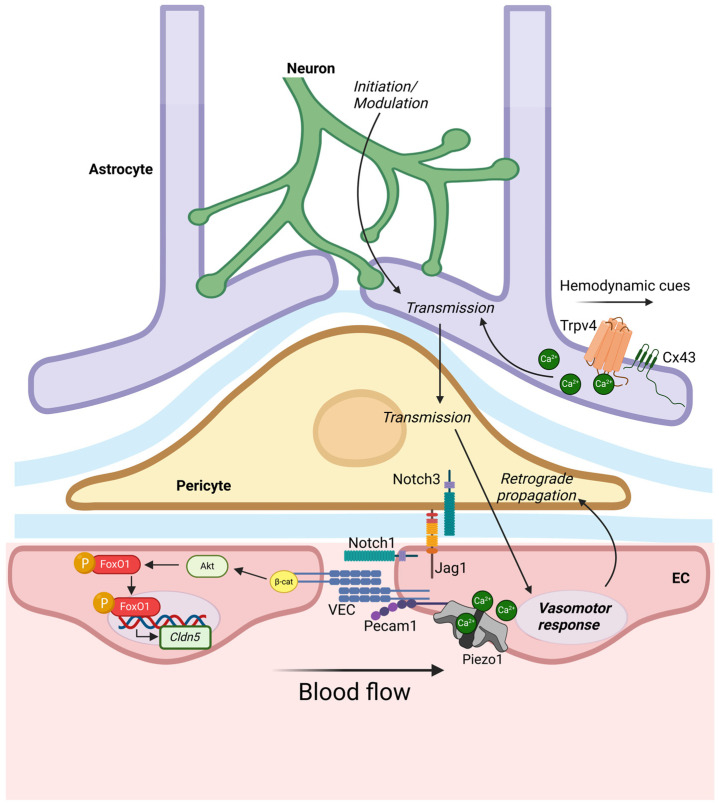
Mechanics of the neurovascular development. During early embryo development, blood flow drives endothelial cell (EC) adhesion by enhancing adherens junction-mediated claudin 5 expression. Likewise, PIEZO1 responds to flow by stabilizing adherens junctions between endothelial cells. The Notch1-Jag1-Notch3 signals encourages EC/pericyte adhesion. During neurovascular coupling, not only chemical but also hemodynamic stimuli activate both neurons and astrocytes. Signals are transmitted to ECs by the pericytes. In ECs, a vasomotor response in generated and transmitted to pericyte, regulating vascular tone and blood pressure. Image created by the BioRender tool.

Endothelial cells also join pericytes through adhesion molecules, including N-cadherin and connexin-43, and by TGFβ−1. Therefore, to better describe the close relation underlying reciprocal regulation during neurovascular development, the concept of “neurovascular unit” (NVU) was introduced during the first Stroke Progress Review Group meeting of the National Institute of Neurological Disorders and Stroke (NINDS) of the National Institutes of Health (NIH) in 2001 (https://www.ninds.nih.gov/About-NINDS/Strategic-Plans-Evaluations/Strategic-Plans/Stroke-Progress-Review-Group, accessed on 24 March 2025). This morpho-functional integration leads neurons and vasculature to reciprocally regulate each other and, in particular, to regulate local perfusion in response to neural activity, in a process known as neurovascular coupling. Neurovascular coupling starts with an *initiation* phase, triggered by hypoxia conditions, chemical mediators as NO, or hemodynamic cues [309,310]. This early signal is dosed during a *modulation* phase, before being transmitted to the astrocytes and, then, to pericytes and ECs surrounding arterioles and capillaries (*neurovascular transmission* phase). In astrocytes, depolarization is promoted by the BK channels [311]. At EC monolayer, neuronal signal is elaborated as a vasomotor response and transmitted to mural cells, responsible for vascular tone and flow regulation (*retrograde propagation/implementation* phases) [312,313]. Neurovascular coupling is driven by hemodynamic forces also in astrocytes. They own mechanosensitive properties mediated by the TRPV4 receptor. Following a decrease in cerebral perfusion pressure, TRPV4/Cx43 coupling results in intracellular Ca^2+^ mobilization, aimed to increase systemic arterial blood pressure and heart rate [314,315,316] (Figure 7).

During neurovascular coupling, ECs respond to hemodynamic cues through the primary cilium. It is a sensory organelle exposed on the apical surface of the ECs and directly linked to the cytoskeletal microtubules, where modulate EC shape, in response to blood flow [317]. During zebrafish development, cilium morphogenesis in brain ECs begins during early vasculogenesis, preceding both flow induction and cardiac contractions, and continues until late-capillaries formation, contributing to vascular integrity. Cilium disruption following shear stress results in increased vessel permeability and intracerebral hemorrhage (ICH) [318]. Moreover, zebrafish mutants for the intraflagellar transport genes exhibit defective cilia and spontaneous ICH, most likely due to impaired sonic hedgehog (shh) signaling [319]. Likewise, Pkd1 mutant mice showed vascular leakage, hemorrhage and failed embryo development [320]. Moreover, flow was shown to activate Notch signal and, downstream, foxc1b in endothelial primary cilia, driving mural cell recruitment to arteries during vessel specification in zebrafish [321]. Together with the primary cilium, PIEZO1 and several TRPV family members drive BBB development and function. PIEZO1 expression is greater in ECs compared to other NVU cell types [322]. Its activation by blood flow during brain vasculature development triggers NOTCH signaling in ECs, enhancing EC/pericyte cross-talk and promoting pericyte proliferation and vessel coverage to ensure BBB properties [323]. By electrophysiological assays, it was shown that ETC branching depends on piezo1-mediated Ca^2+^ flux and *piezo1* loss of function causes branch retraction in zebrafish larvae [324]. Calpain triggers ETC branch retraction downstream Ca^2+^ transient perturbation. In contrast, NOS regulates ETC branch extension [325]. In the same study, Liu and colleagues demonstrated that *piezo1* mutant zebrafish larvae show enlarged brain vessels and increased number of vascular segments, suggesting the pivotal role of this mechanosensitive channel in early angiogenesis. In adulthood, ECs of brain arteries, veins and capillaries express PIEZO1, that remains responsive to blood flow and pressure. In addition, it induces NO-mediated vasodilatation in small diameter vessels, upon activation by blood cells. A similar response is observed in ECs, following neuronal depolarization [326]. In the retinal vasculature, Piezo1 was shown to colocalize with Pecam1 at AJs. Under shear stress conditions, Piezo1 over-activation disrupts Pecam1 structural organization and drives Cdh5 recruitment at AJs to maintain BBB integrity [122]. beyond its role in blood vasculature, Piezo1 further drives development of meningeal lymphatic vessels, where it regulates cerebrospinal fluid drainage. *Piezo1* loss of function results in slower drainage and ventricular fluid accumulation [327]. In the CNS, also neurons express PIEZO1. Here, it contributes to neuroinflammation after ICH. In particular, *Piezo1* expression increases following ICH, when promotes IL-6 production and NLRP3-mediated inflammasome activation [328]. Conversely, Piezo1 block decreases Bcl2-mediated neuronal apoptosis and reduces brain edema by down-regulating *AQP4* expression, suggesting as PIEZO1 inhibition can contribute to neuronal tissue rescue, in an ICH mouse model [329].

Among the TRP family, TRPM4, TRPC3, TRPC6 and TRPA1 are involved in myogenic constriction and arteriolar contractility, regulating cerebral blood flow in response to pressure [330,331,332]. The TRPV members mainly regulate neurovascular coupling. TRPV2 is the most well characterized TRP channel in brain ECs, where it controls proliferation, migration and tubulogenesis [333]. Recently, Ramos et al. demonstrated heterozygous *TrpV2* mutant rats exhibit enlarged retinal vessels, covered by thinner endothelium and increased inflammatory and oxidative marker expression, suggesting that this MC may play a protective role in diabetic retinopathy [334]. *TRPV4* is expressed in all neurovascular cell types. In arteriolar and capillary ECs, it controls vasodilatation. In detail, in arterioles TRPV4-induced relaxation requires the activation of the endothelial muscarinic acetylcholine receptor and, for this reason, its role in vascular dementia onset is strongly hypothesized [335,336]. Moreover, when activated after ischemic stroke, TRPV4 enhances brain EC proliferation, angiogenesis and neurogenesis, thereby restoring brain functional properties [337,338]. In contrast, microglia-derived TNF-α induces increased TRPV4 expression in multiple sclerosis. In this context, TRPV4 contributes to BBB damage by increasing proinflammatory cytokine production and by down-regulating *Cldn5*, *Cdh5* and *Tjp1* expression [339]. Finally, in brain vasculature, TRPV1 controls vessel permeability and its expression increases after ischemic damage, contributing to brain edema, EC apoptosis and neuroinflammation [340,341].

## 6. Hemodynamics in Pathological Vascular Phenotypes

Perturbed hemodynamics can result in vasculature defects. For instance, the role of ENG and ALK1 as mechanosensors and regulators of vessel diameter was previously described [230]. They are BMP receptors and germline loss of function mutations in their coding genes cause the autosomal dominant hereditary hemorrhagic telangiectasia (HHT) phenotype, also known as Osler-Weber-Rendu syndrome [342]. Patients with HHT show focal vascular lesions featured by direct arteriovenous shunt, due to the lack of the capillary bed, and so called arteriovenous malformations (AVM). Sporadic AVM can also occur due to impaired expression of endothelial differentiation markers [343,344,345]. ALK1 controls arteriovenous specification and EC polarization against blood flow direction, by mediating integrin signaling with VEGFR2. Downstream, YAP/TAZ nuclear translocation controls cell migration [346]. Evidence of disturbed shear stress in HHT development was proposed by Baeyens et al., based on studies performed on mouse retina. In detail, they showed that BMP expression increased under shear stress condition and this condition enhanced Alk1-Eng coupling. Downstream, this response resulted in EC proliferation arrest and mural cell recruitment, suggesting that this mechanism contributes to EC specification and vascular stability maintaining. Therefore, perturbation of this signal may contribute to the HHT phenotype [347]. In addition, it was recently shown that shear stress induces BMP9/Alk1 interaction and, downstream, the activation of SMAD1/5 cascade, both in-vitro and in-vivo. Perturbation of this cascade results in the AVM phenotype. More in detail, SMAD1/5 signal activates the transcription of the *GJA4* gene, encoding for the connexin 37 (Cx37). Cx37 was shown to negatively control EC migration in response to blood flow, suggesting its role in shunt regulation [348].

Together with HHT, also pulmonary arterial hypertension (PAH) arises following perturbation of TGF-β signal and, in particular, due to *BMPR2* gene mutation, encoding for the BMP type 2 receptor. In PAH, *BMPR2* loss of function mutations result in permeable lung vessels and increased pulmonary artery pressure, due to amplification of TGFβ-SMAD signaling [349]. However, mechanobiological factors, comprising EC contractile phenotype, ECM remodeling and stiffness, also contribute to the phenotype. In this field, formation of mixed canonical TGFβ-SMAD2/3 and lateral TGFβ-SMAD1/5 complexes was shown in-vitro, upon *BMPR2* loss of function. As a consequence, ECs undergo EndMT, down-expressing endothelial specific markers as *PECAM1* and *CDH5*. In contrast, increased expression of β1-integrin, integrin-linked kinase and fibrillin-1 was reported, suggesting BMPR2 as antagonist of the TGFβ-SMAD signaling, in response to ECM and AJ stiffness [350].

Again, TGF-β1 activation in stiffer matrices results in valve stiffening, a condition in which valvular ECs undergo EndMT, leading to aortic valve stenosis. However, in this case, downstream signal involves the Wnt/β-catenin pathway [351].

## 7. Discussion

Discovery of cell ability to be responsive to mechanical cues allowed the recent development of a novel research field known as mechanobiology. Broadly, mechanobiology encompasses all mechanisms and proteins involved in mechanotransduction, defined as the process by which a cell converts mechanical forces into biological responses. External mechanical stimuli that drive cell fate include, but are not limited to, ECM stiffness, rigidity, density, hemodynamic forces, flow direction, hydrostatic pressure [4,147,220]. Likewise, adherens junctions ensure the transmission of cytoskeletal mechanics between adjacent cells and, in this field, tension and compression are the most common forces [8].

Mechanosensitive proteins include focal adhesion proteins and mechanosensitive ion channels. The large number of mechanotransducers are heterogeneously expressed in different tissues, making cells responsive to specific mechanical stimuli. For this reason, the mechanical control of cell fate has been shown to be crucial during both development and remodeling phenomena. During development, ECM forces control mesoderm specification, gastrulation and morphogenetic movements by activating integrins and cadherins [166]. Organogenesis, in turn, further requires the activation of mechanosensitive channels, resulting in their gating and ion influx within the cell. In this context, the TRP and PIEZO families are the most well characterized. Notably, the role of polycystins in primary cilium and kidney development has been largely studied, showing as PKD1 and PKD2 mutations result in the autosomal dominant polycystic kidney disease [104,105,106]. Likewise, PIEZO1 involvement in muscle and bone development was confirmed. More in detail, in bone tissue PIEZO1 activation by blood flow induces tissue repair after fracture [202].

However, mechanical control of cell function is even more evident during vascular development, when hemodynamic cues drive EC differentiation, polarization and migration. In this context, receptors for soluble growth factors also act as mechanosensors [217,230]. The endothelial cell response to blood flow further involves the VEGF signaling and promotes cell adhesion. In particular, this phenomenon is crucial during BBB development, when expression of TJ proteins is promoted by VEC-mediated cell adhesion, when blood starts to flow [306,307,308].

The importance of mechanosensation is proven by pathological phenotypes arising due to mutations in genes encoding mechanoreceptors. Among these, failed mechanotransduction was linked to fibrosis and congenital heart defects [352,353,354]. For this reason, targeting mechanosensors is a novel and promising therapeutic strategy in the field of regenerative medicine.

## 8. Conclusions

Cell responses to external mechanical cues drive intracellular biological processes aimed to control tissue development, remodeling and physio-pathological conditions. In the last few years, attention to cell mechanotransduction has been rapidly increasing, due to the large number of biological processes regulated by mechanical stimuli. As discussed, proteins acting as mechanosensor include focal adhesion components as integrins, vinculin and cytoskeleton elements, responsive to ECM mechanical properties. The discovery of epigenetic regulation as a final event of mechanotransduction is increasing perspectives on the possibility of regulating gene expression by modulating ECM composition, stiffness and rigidity. This strategy is currently considered very promising for tissue regeneration. On the other hand, the identification of ion channels that gain gating activity upon mechanical stimulation has clarified several aspects of development and remodeling in various tissues, including bone, teeth, muscle and vessels. In the field of vascular biology, mechanotransduction drives endothelial differentiation and vessel morphogenesis since the early stages of development, as mechanosensor expression precedes blood flow. In particular, PIEZO1 and TRPV4 mechanosensitive ion channels control endothelial progenitor cell differentiation and capillary plexus formation. Interestingly, defects in mechanotransduction result in aberrant angiogenetic sprouting due to impaired endothelial tip/stalk phenotype acquisition. In addition, blood flow and shears stress promote cell adhesion and pericyte recruitment during BBB development, and aberrant mechanotransduction has been confirmed to result in enlarged vessels and aneurysm. Recent studies aim to develop modulable mechanofluidic devices to control fluid dynamics in-vitro, for endothelial and myocardial regeneration after injury.

## Figures and Tables

**Table 1:** Mechanosensitive ion channels in mammalian cells. The table lists the main classes of proteins acting as MCs in mammalian cells. For each family (Column I), the activating stimuli (Column II), the intracellular effector (Column III) and the biological response within the cell (Column IV) are listed. References are indicated in the reference list. BK: big potassium; CaM: calmodulin; ENaC: epithelial sodium channel; GPCR: G-protein coupled receptor; NO: nitric oxide; TREK: TWIK-Related K^+^; TRP: transient receptor potential.

Mechanosensor	Stimulus	Downstream Effector	Cell Response	Reference
ECM-integrin-cytoskeleton proteins	Stiffness, tension,	KLF2/4, YAP/TAZ	Differentiation, apoptosis, proliferation	[52]
ENaC	Pressure, shear forces	NO	Neurosensation, blood pressure regulation	[53,54]
TREK	Membrane tension	AKAP150, β-COP, Mtap2, sortilin	K^+^ flow regulation	[55,56]
BK	Membrane electric potential	K^+^	Cell hyperpolarization, smooth muscle tone	[57]
GPCRs	Shear stress, mechanical stretch	Heterogeneous	Cell adhesion	[58]
TRPC	Oxidative damage	Calcineurin	Memory, LTP, pain	[59,60]
TRPM	Oxidative stress, inflammation, temperature	Heterogeneous	Hormone release, apoptosis	[61,62]
TRPV	Mechanical and osmolar stimuli	Phosphatidyl-inositol cascade	ECM remodelling	[63]
TRPA	Mechanical stress	Ca^2+^	Nociception, temperature sensation	[64]
TRPML	Membrane tubulation, pH	CaM, calcineurin	Autophagy	[65]
TRPP	Stiffness, cell tension, shear stress	Ca^2+^	Tubule morphogenesis	[66]
PIEZO1	Shear stress, stiffness	KLF2/4, YAP/TAZ	Vessel morphogenesis, bone repair	[67]
PIEZO2	Touch, shear forces, stretch	S1P, ERK	Sensory neuron depolarization	[68]

## Abbreviations

The following abbreviations are used in this manuscript:

AJ	Adherens Junction
AVM	ArterioVenous Malformation
BBB	Blood-Brain Barrier
BK	Big Potassium
BMP	Bone Morphogenetic Protein
CNS	Central Nervous System
EC	Endothelial Cell
ECM	Extracellular Matrix
ENaC	Epithelial Sodium Channel
EndMT	Endothelial-to-Mesenchymal Transition
ENG	Endoglin
ETC	Endothelial Tip Cell
FAK	Focal Adhesion Kinase
GPCR	G-Protein Coupled Receptor
HHT	Hereditary Hemorrhagic Telangiectasia
ICH	Intracerebral Haemorrhage
KLF	Krüppel-Like Factor
LINC	Linker of Nucleoskeleton and Cytoskeleton
LTP	Long Term Potentiation
MC	Mechanosensitive Channel
MSC	Mesenchymal Stem Cell
NO	Nitric Oxide
NVU	NeuroVascular Unit
SOCE	Store Operated Ca^2+^ Entry
TAZ	Transcriptional coactivator with PDZ-binding motif
TJ	Tight junction
TREK	TWIK-Related K^+^
TRP	Transient Receptor Potential
VEC	Vascular Endothelial Cadherin
VEGF	Vascular Endothelial Growth Factor
VSMC	Vascular Smooth Muscle Cell
YAP	Yes-Associated Protein
